# Age Differences in Preferences for Fear-Enhancing Versus Fear-Reducing News in a Global Pandemic

**DOI:** 10.1093/geroni/igaa057.3417

**Published:** 2020-12-16

**Authors:** Anthony Villalba, Jennifer Stanley, Jennifer Turner, Michael Vale

**Affiliations:** The University of Akron, Akron, Ohio, United States

## Abstract

Older adults (OA) prefer positive over negative information in a lab setting, compared to young adults (i.e., positivity effects; YA). The extent to which OA avoid negative events or information relevant for their health and safety is not clear. We first investigated age differences in preferences for fear-enhancing versus fear-reducing news articles during the Ebola Outbreak of 2014. We built upon this pilot study to further investigate this research question during the COVID-19 pandemic. In this study, 164 YA (18-30 years) and 171 OA (60-80 years) responded to an online survey about their preferences, feelings, and behaviors related to the COVID-19 pandemic across 13-days during the initial peak of the pandemic in the United States. Both YA and OA preferred to read positive over negative news about the coronavirus, but OA were even more likely than YA to prefer the positive news article. No age differences in the fear of contraction were found, but OA engaged in more health-protective behaviors compared to YA. Additionally, media engagement was related to fear for both age groups, with social media engagement, specifically, emerging as a key moderating factor for protective behavior change. Although OA may not fear or seek out negative information related to a health concern; they still engage in more protective health behaviors compared to YA. In this study, positivity effects are shown to exist within a health-related event, but OA appeared to still attend to enough negative information about COVID-19 to avoid impairing their health protective behaviors.

